# Quadriceps Strength and Temporal Preparation in Elderly Adults: The Mediating Role of Beta Oscillation

**DOI:** 10.1111/ejn.70101

**Published:** 2025-04-01

**Authors:** Ming‐Cho Ho, Hao‐Lun Fu, Shih‐Chun Kao, David Moreau, Wei‐Kuang Liang, Hsin‐Yu Kuo, Chun‐Hao Wang

**Affiliations:** ^1^ Institute of Physical Education, Health & Leisure Studies National Cheng Kung University Tainan City Taiwan (ROC); ^2^ Department of Physical Therapy Tzu Hui Institute of Technology Pingtung County Taiwan (ROC); ^3^ Wellcome Centre for Integrative Neuroimaging, FMRIB, Nuffield Department of Clinical Neurosciences University of Oxford Oxford UK; ^4^ Department of Psychology National Cheng Kung University Tainan City Taiwan (ROC); ^5^ Department of Health and Kinesiology Purdue University West Lafayette Indiana USA; ^6^ School of Psychology University of Auckland Auckland New Zealand; ^7^ Centre for Brain Research University of Auckland Auckland New Zealand; ^8^ Institute of Cognitive Neuroscience National Central University Taoyuan City Taiwan (ROC); ^9^ Department of Internal Medicine National Cheng Kung University Hospital, College of Medicine, National Cheng Kung University Tainan City Taiwan (ROC)

**Keywords:** cognitive aging, foreperiod effect, muscle strength, neural oscillation, response preparation

## Abstract

This study investigated the relationship between lower limb muscle strength and temporal preparation in older adults using an electroencephalogram to assess neural oscillations during cognitive processes. Forty older adults were divided into higher (HSG, 70.40 ± 5.15 years) and lower muscle strength (LSG, 71.43 ± 4.86 years) groups based on quadriceps strength estimated via a manual muscle test. Functional mobility was assessed using the Timed Up and Go (TUG) test, while temporal preparation was evaluated using a choice response time (RT) task with randomly varying foreperiods (FPs) that required lower limb motor responses. The HSG outperformed the LSG on both the TUG test (HSG: 6.07 ± 1.14 vs. LSG: 6.79 ± 0.88, *p* = 0.031) and the cognitive task (HSG: 462.97 ± 51.06 ms vs. LSG: 525.86 ± 73.69 ms, *p* = 0.002), despite no clear FP effect in either group. Additionally, the HSG demonstrated a more pronounced modulation of oscillatory beta power during the late phase of longer FP trials (*qs* < 0.05, FDR corrected), whereas no significant modulation was observed during shorter FP trials. Crucially, mediation analysis indicated that beta power significantly mediated the relationship between lower limb strength and RT in longer FP trials [*b* = −24.21; 95% CI = (−53.51, −0.24)]. In summary, these findings suggest that lower limb strength may influence the development of temporal preparation during longer preparatory periods by modulating beta power, potentially serving as a compensatory mechanism to mitigate age‐related declines in cognitive processing speed and preserve functional mobility.

## Graphical abstract

This study demonstrates that greater lower limb strength is associated with stronger beta power modulation during cognitive processing, which mediates the relationship between strength and task performance in elderly adults. These findings highlight the potential of maintaining lower limb strength to support neural activity and enhance functional mobility, potentially reducing fall risk.

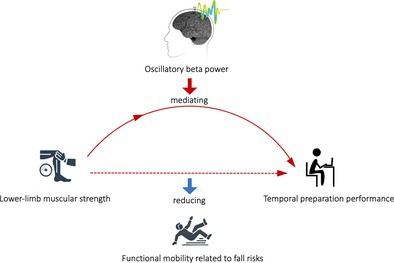

**Abbreviations**
BDI‐II: Beck Depression Inventory‐II
EEG: electroencephalogram
EMG: electromyography
FP: foreperiod
HSG: higher muscle strength group
IS: imperative signal
LSG: lower muscle strength group
MMSE: Mini‐Mental State Examination
MMT: manual muscle test
MoCA: Montreal Cognitive Assessment
RT: response time
TUG: Timed Up and Go
WS: warning signal

## 1 Introduction

Aging has been associated with an increased risk of deteriorating neurophysiological functions, which frequently results in decreased cognitive function (Deary et al. 2009). Crucially, declines in specific cognitive functions such as processing speed and executive function have been associated with a higher risk of injurious falls in elderly adults (Welmer et al. 2017), which can further lead to severe outcomes including prolonged pain, functional limitations, disability, and even death (Kannus et al. 2005). Accordingly, maintaining cognitive function in the elderly to mitigate the risk of fall has become a significant issue (Krivanek et al. 2021).

Age‐related decreases in processing speed are key indicators of cognitive decline (Godefroy et al. 2010) and many cognitive abilities in older adults can be effectively assessed through measures of processing speed, such as response time (RT) (Eckert 2011; Yang et al. 2019). In this regard, in a large‐scale population‐based study of older adults, slow processing speed has been suggested as a potential early marker of declining mobility and global cognition (Welmer et al. 2014). Moreover, a subsequent study by Welmer et al. (2017) found that deficits in processing speed and executive function were associated with an increased risk of injurious falls over a longer follow‐up period, particularly among individuals without cognitive impairment. In support of this, Davis et al. (2017) reported that slow processing speed was the strongest and most consistent predictor of future falls, compared with both physical and emotional functioning. One plausible explanation for the association between processing speed and falls is that slower processing speed may impair an individual's ability to react effectively to environmental challenges, increasing the likelihood of falls (Kearney et al. 2013; Welmer et al. 2017). Therefore, examining RT during cognitive processing may offer valuable insights into the cognitive mechanisms underlying fall risk in aging populations.

Notably, RT is influenced not only by the cognitive processes required for task execution but also by preparatory mechanisms that help establish and maintain an optimal processing state before a response. Preparatory mechanisms encompass a range of processes, including anticipatory postural adjustments (MacKinnon et al. 2007), attentional focus (Battistoni et al. 2017), and motor planning (Svoboda and Li 2018), which collectively help establish an optimal state for executing timely and accurate responses. However, age‐related declines in prefrontal cortex function (Vallesi et al. 2009) and reduced dopamine levels (Volkow et al. 1998) may impair the ability to maintain an optimal preparatory state, leading to slower RTs and less efficient motor responses. Therefore, the ability to prepare effectively may partially account for age‐related cognitive slowing and the increased risk of falls in older adults. Experimentally, preparatory processes can be observed in RT tasks that include a warning signal (WS) followed by an imperative signal (IS) (Nobre et al. 2007). If the WS provides predictive value regarding the IS's occurrence (e.g., explicit temporal or spatial cues), the preparatory process may be activated and significantly influence RT performance. Specifically, preparation can be either specific or nonspecific, depending on whether it involves a predetermined response to a specific stimulus. When the upcoming response is unknown, only temporal preparation affects RT, which is also referred to as nonspecific preparation. A common example of temporal preparation in daily life is waiting at a red traffic light. You may not know the exact moment it will turn green, but as more time passes, your readiness to press the accelerator increases, demonstrating an implicit buildup of preparatory activation.

Temporal preparation refers to the ability to covertly use temporal information to synchronize actions with appropriate timing (Nobre et al. 2007; Wang et al. 2013), which can be examined by manipulating the parameters of a foreperiod (FP) between a WS and an IS in an RT task (Niemi and Näätänen 1981). Typically, when different FP lengths are interleaved within a block, RTs are shorter when the FP is longer, also referred to as the variable FP effect (Niemi and Näätänen 1981), which has been interpreted as a modulation of conditional probability (i.e., the likelihood or temporal uncertainty of the signal) (Niemi and Näätänen 1981; Nobre and Coull 2011). This increase in certainty about the timing of the IS is reflected in faster RTs, likely as a result of enhanced preparedness. For example, consider using two FPs of 500 and 1500 ms, each occurring randomly but with equal frequency within a block. In a given trial, the probability that the IS will occur is 0.5 for both the 500‐ms FP and the 1500‐ms FP. If no signal occurs after 500 ms, the probability that it will occur at 1500 ms becomes 1, resulting in faster RT as the FP increases. Thus, the ability to show faster RT during the long FP condition relative to the short FP condition has been associated with an endogenous and strategic process (Niemi and Näätänen 1981).

Previous research on temporal preparation in elderly adults during variable FP tasks has shown inconsistent findings. Bherer and Belleville (2004), using a simple RT task with variable FP, observed that elderly adults generally exhibited slower RTs and a larger FP effect compared to young adults, primarily because of disproportionate slowing at the shortest FP. This suggests that elderly adults may be less likely to prepare during periods of high temporal uncertainty, probably because focusing on the entire set of FPs may be too demanding for them. In contrast to this finding is the lack of an FP effect, as observed by Vallesi et al. (2009). In their study, they compared RT performance in a choice task with variable FPs between young and elderly adults, showing that elderly adults generally exhibited slower RTs and did not show the variable FP effect observed in younger adults. This pattern suggests not only cognitive slowing but also a specific impairment in the elderly's ability to monitor the temporal dynamics of changing conditional probabilities during the preparatory process. Given the converging evidence from children (Vallesi and Shallice 2007), patients with brain lesions (Vallesi et al. 2007), and neuroimaging research (Vallesi et al. 2009), which have shown that temporal preparation primarily relies on the integrity of the prefrontal cortex—a region known to be vulnerable to cognitive aging (West 1996), examining the ability of elderly adults to prepare a response during variable FP tasks may provide valuable insights into the mechanisms of age‐related cognitive processing speed.

Regular physical activity and increased physical fitness have been associated with improved neurocognitive function in elderly adults (Dupuy et al. 2024; Erickson et al. 2022; Kramer et al. 1999; Wang 2020; Wang and Tsai 2016). A few studies have also observed a positive effect of higher aerobic fitness on temporal preparation in elderly adults. In a study using both simple and choice RT tasks with variable FPs, Renaud, Bherer, et al. (2010) found that elderly adults with higher levels of fitness performed better in the shortest FP (i.e., 1 s) within the short temporal window condition (i.e., 1, 3, and 5 s) and in the longer FPs (i.e., 5 s and 7 s) within the long temporal window condition (i.e., 5, 7, and 9 s). These findings suggest that higher levels of aerobic fitness may be associated with an enhanced ability to rapidly develop an optimal preparatory state for unlikely events, as well as a better capacity to sustain this state over longer delays. Moreover, Renaud, Maquestiaux, et al. (2010) demonstrated that sedentary elderly adults improved both their aerobic fitness levels and temporal preparation following a 12‐week aerobic exercise intervention. Specifically, they observed that this beneficial effect was more pronounced for RTs during longer FPs, suggesting an enhanced ability to utilize stimulus probability and maintain a preparatory state over extended periods.

Despite the well‐documented positive effects of aerobic fitness, it is important to consider the role of lower limb muscular strength in cognitive functioning among older adults (Chen et al. 2015), which appears to be independent of aerobic fitness (Scherder et al. 2010). Notably, previous research has demonstrated that upper extremity strength (e.g., grip strength) is not associated with injurious falls (Welmer et al. 2017), suggesting that assessments focusing on lower extremity function may provide a more sensitive measure for evaluating factors related to fall risk in older adults. Indeed, stronger muscular strength, particularly in the lower limbs, may be related to greater temporal preparation through improved neuromuscular efficiency (Blackburn et al. 2000; Kuruganti et al. 2006), such as faster motor unit recruitment and enhanced proprioceptive feedback. These mechanisms could facilitate more precise and timely motor responses, which are critical for tasks requiring rapid adjustments, such as maintaining balance or avoiding obstacles. Therefore, we propose that muscular strength may influence temporal preparation through different mechanisms from the cardiovascular and cerebrovascular mechanisms associated with aerobic fitness. In this context, quadriceps strength has been identified as a key indicator of both fall risk (Ahmadiahangar et al. 2018) and the ability to perform daily activities (Wearing et al. 2019), highlighting its crucial role in maintaining functional independence. However, it remains unclear whether temporal preparation, particularly for lower limb responses, is related to lower limb muscular strength. Interestingly, previous research using a spatial cueing task involving the lower limbs has shown that children with poor motor coordination exhibit attentional deficits during the preparatory period, as evidenced by slower lower limb responses (Wang, Lo, et al. 2015). This finding may be attributed to the sensitivity of foot performance tests to motor control problems (Peters 1988). It is thus speculated that investigating lower limb responses could enhance our understanding of temporal preparation and functional mobility related to fall risk in elderly adults.

In addition to investigating temporal preparation for lower limb responses in elderly adults, we also evaluated task‐related changes in oscillatory electroencephalogram (EEG) activity using time–frequency analysis approaches (Roach and Mathalon 2008) to probe the underlying neural mechanisms. This approach enables one to investigate event‐related changes in the magnitude (i.e., power) of EEG oscillations at specific frequencies over time relative to events of interest (Makeig et al. 2004). Such analysis is particularly valuable for studying cognitive processes as they unfold over time (Rohenkohl and Nobre 2011). Past research using time–frequency EEG analysis has demonstrated that theta (4–7 Hz) and beta (15–35 Hz) power over the central‐midline area reflect response preparation during periods of uncertainty (Cravo et al. 2011; Tzagarakis et al. 2010). Specifically, theta power oscillation has been shown to vary with temporal expectation, potentially serving as an attentional mechanism for temporal preparation (Cravo et al. 2011). On the other hand, beta power has been found to be associated with the modulation of RT differences related to motor preparation (Tzagarakis et al. 2010; Wang, Liang, et al. 2015). Thus, these EEG oscillations may offer insights into the differential influences of lower limb muscular strength on temporal preparation at both attentional and motor levels.

Taken together, the present study aimed to investigate whether elderly adults with different levels of lower limb muscle strength exhibit performance differences in nonspecific temporal preparation for lower limb responses and the associated risk of falls. To address this, we used the Timed Up and Go (TUG) test to evaluate the functional mobility associated with fall risk and a variable FP paradigm to measure their temporal preparation. Additionally, we measured EEG oscillations related to temporal preparation to shed light on the underlying neural mechanisms. If lower limb muscle strength is related to functional mobility and temporal preparation, it is hypothesized that elderly adults with greater lower limb strength will demonstrate better performance on the TUG test and in the variable FP paradigm. Furthermore, we anticipated that elderly adults with different levels of lower limb strength would exhibit differential modulation of neural oscillations related to temporal preparation. Additionally, if lower limb strength is linked to both EEG oscillations and temporal preparation, we aimed to explore whether the influence of lower limb strength on temporal preparation could be mediated by its effect on EEG oscillations. The findings of this study are expected to enhance our understanding of the cognitive and neural mechanisms underlying temporal preparation for lower limb responses in elderly adults. Moreover, this study provides evidence supporting the importance of maintaining higher levels of lower limb muscular strength for neurocognitive health and functional mobility, potentially contributing to a reduced risk of falls in this population.

## 2 Material and Methods

### 2.1 Participants

The initial cohort consisted of 65 participants aged 65–84 years from Tainan City, Taiwan. Following demographic assessments and questionnaire screenings, 18 participants were excluded because of neurological disorders, cardiovascular diseases, psychiatric conditions, or an inability to follow the experimental procedures. The remaining 47 eligible participants then underwent cognitive testing and EEG recording. However, because of incomplete EEG recordings or technical issues, such as excessive noise in the EEG signals, an additional seven participants were excluded, resulting in a final sample of 40 participants. The final cohort comprised 40 healthy older adults aged 65–81 years. All participants were screened for cognitive impairment and depression using the Montreal Cognitive Assessment (MoCA; > 26 points) (Nasreddine et al. 2005), Mini‐Mental State Examination (MMSE; > 24 points) (Folstein et al. 1975), and Beck Depression Inventory‐II (BDI‐II; < 14 points) (Beck et al. 1996), indicating no signs of cognitive impairment or depression. These participants were reported to be free of neurological problems, cardiovascular diseases, and medications that could affect cognitive function. Informed consent was obtained from all participants before the study began, and the study was approved by the Human Research Ethics Committee of National Cheng Kung University.

### 2.2 Procedures

To eliminate the possibility of biases influencing the experimental results, the participants were not informed of the purpose of the study. The experimenters also ensured that the participants did not engage in physical activity on the day before the study to prevent any potential acute effects of physical activity (Kao et al. 2022, 2020; Moreau and Chou 2019; Wang et al. 2023). This study consisted of three phases: (1) completion of questionnaires, including a demographic questionnaire, MoCA, MMSE, and BDI; (2) lower limb muscle strength and functional mobility assessments, using the manual muscle test (MMT) and the TUG test; and (3) the cognitive task (i.e., the variable FP paradigm) with concurrent EEG recording. All participants first visited our lab for Phases 1 and 2 to confirm their eligibility, which took approximately 30 min. Within a week, eligible participants were invited for a second visit to complete the cognitive task and EEG recording, which took about 40 min.

### 2.3 Manual Muscle Test Measurement

The study employed MMT, a commonly employed clinical tool by experienced physical therapists to assess lower limb muscle strength (Cuthbert and Goodheart 2007). Considering the significance of quadriceps strength as an essential measure of cognitive performance (Chen et al. 2015), falls (Ahmadiahangar et al. 2018), and daily activities (Wearing et al. 2019) in elderly adults, this study adopted the quadriceps as the primary grouping indicator for lower limb muscle strength. The MMT method was administered by a physical therapist with 8 years of clinical experience. Participants were seated on a treatment table with their hands by their sides to ensure stability. They were allowed to lean back slightly to maintain tension in their hamstrings but were instructed not to fully extend their knees. The therapist positioned themselves beside the foot being tested, placing one hand on the participant's thigh and applying force above the ankle joint on the calf with the other hand. The measurement involved the participant extending their knee joint without fully reaching 0°. The following instruction was given: “Please straighten your knees, hold the position, and resist being pushed down by my hand.” Performance was assessed using standardized testing positions (Hislop et al. 2014; Mathur et al. 2019), with a six‐point grading scale. To elaborate, this scale ranges from 0, indicating no visible or palpable muscle contraction, to “Grade 3,” where the limb can be moved against gravity without resistance, and “Grade 5,” indicating that the participant can contract their muscle against full manual resistance applied by the tester.

According to previous research (Fosang and Baker 2006), the difference in hip and knee muscle strength between individuals with an MMT score of 3 and those with a score of 5 was examined during a walking test. The results showed that muscle groups with a score of 3 produced significantly lower maximum torque output during ambulation compared to those with a score of 5. Based on these findings, this study classified participants into two groups: the higher muscle strength group (HSG), consisting of individuals with an average score of both dominant and nondominant legs above 4, and the lower muscle strength group (LSG), comprising those with an average score of 3.5 or below.

### 2.4 TUG

This study used the TUG test to assess participants' functional mobility related to fall risk. The intratester and intertester reliability of the TUG test in elderly populations has been reported to be high, with the intraclass correlation coefficient values ranging from 0.92 to 0.99, whereas reliability in community‐dwelling populations has been found to be moderate (0.56) (Steffen et al. 2002). Additionally, recent findings have shown a significant correlation between the TUG test and various balance markers measured by the OptoGait system (Nightingale et al. 2019), further validating its use as a tool for screening balance deficits that relate to increased fall risk in older adults. The test followed the standard protocol, which involves the participant standing up from a seated position, walking around a triangular cone at a comfortable speed, and then sitting back down in the chair. The test was administered twice, and the best performance was recorded for analysis (Sedaghati et al. 2022).

### 2.5 Variable FP Paradigm

The current study utilized a variable FP paradigm, programmed with E‐prime 2.0, to assess participants' ability to temporally prepare for a stepping response while concurrently recording EEG data. Given that prior research has shown that the influence of physical exercise or fitness on temporal preparation is more pronounced in choice RT tasks than in simple RT tasks (Renaud, Maquestiaux, et al. 2010), this study employed the variable FP paradigm using a choice RT design. Notably, here we used only two FPs to increase the conditional probability of stimulus occurrence for the shortest FP. This manipulation is crucial to prevent misleading age‐related differences, as elderly adults tend not to prepare a response in advance for a short FP with high temporal uncertainty (Bherer and Belleville 2004; Vallesi et al. 2009).

**FIGURE 1:**
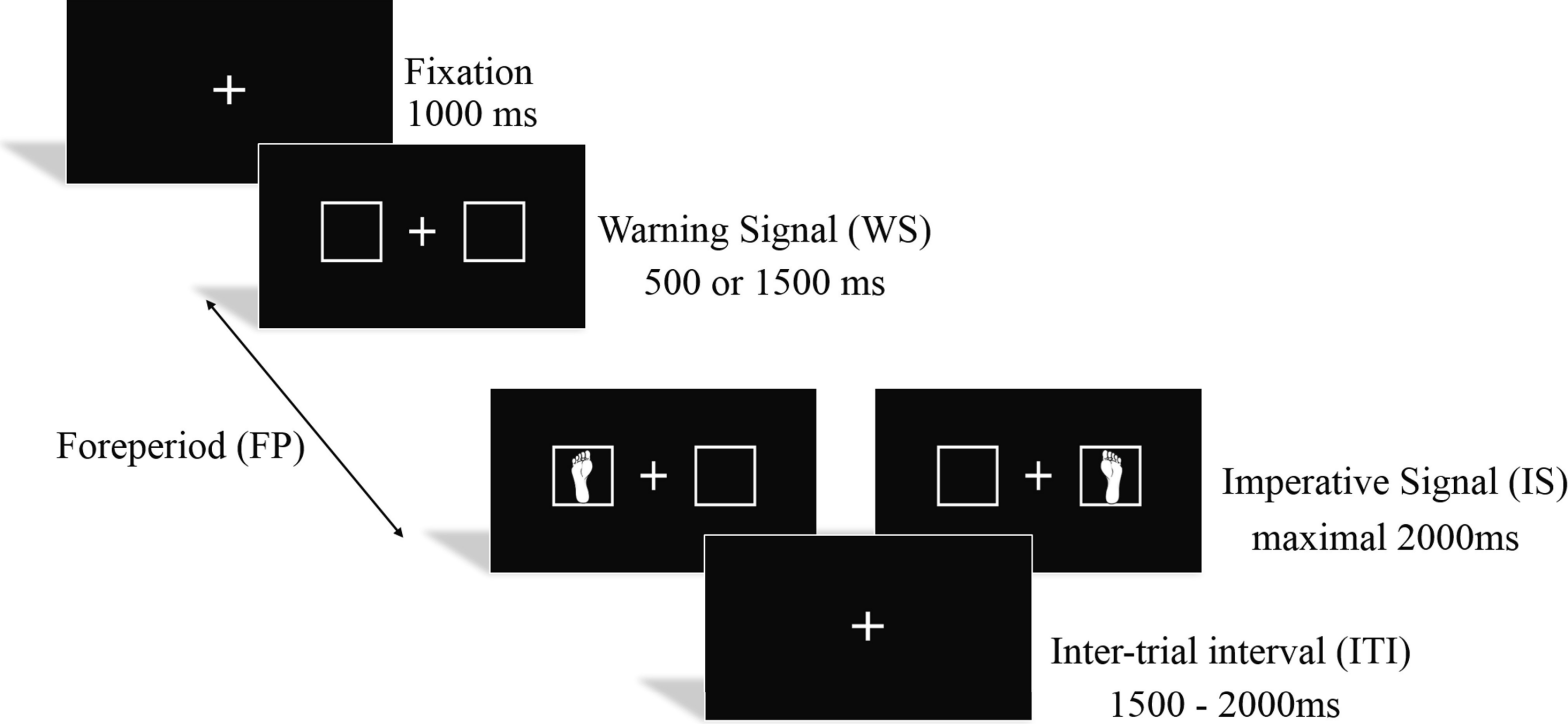
Illustration of the variable FP paradigm. The FP between the WS and the IS was either 500 or 1500 ms.

This cognitive test was conducted in a dimly lit and soundproof room, with participants seated in front of a screen positioned at eye level and approximately 100 cm away. The procedure of the paradigm is illustrated in Figure 1. The WS consisted of a white fixation point flanked by two square boxes, which was followed by the IS (i.e., a footprint) after a randomly determined FP of either 500 ms (FP500) or 1500 ms (FP1500) with equal probability. This range of FPs has been commonly used in research investigating the mechanisms of temporal preparation (Niemi and Näätänen 1981; Wang et al. 2013). The visual stimuli were set at a height of 0.93 cm and a width of 0.91 cm for the cross and 1.8 cm × 1.8 cm for the square boxes, which were displayed on a 21‐in. LED monitor against a black background.

To assess the RTs of the lower limb response during the cognitive paradigm, participants were required to step on the corresponding foot pedal according to the position of the footprints presented in the square box (Hsieh et al. 2025). To enhance response execution, foot pedals were positioned on the left and right sides of the participants' feet and equipped with antislip pads. RT performance was automatically recorded by the E‐Prime system and defined as the time interval from stimulus onset to the completion of movement. Prior to the formal test, participants completed a practice block of 12 trials to familiarize themselves with the task procedure. The formal task consisted of four blocks, with each round comprising 52 trials, resulting in a total of 208 trials. Behavioral and EEG data were recorded during task completion. Participants were instructed to respond as accurately and quickly as possible while minimizing discomfort, and they were also reminded to avoid making saccades during the presentation of laterally presented stimuli.

### 2.6 EEG Acquisition

The EEG recording procedure was in accordance with established protocols of our previous studies (Wang et al. 2017), which was conducted using a Nu‐Amps EEG amplifier and the Scan 4.3 software (Neuroscan Inc., El Paso, TX, USA) with 32 electrodes affixed to an elastic cap (Quick‐Cap; Compumedics, Neuroscan Inc.) configured to the International 10–20 System. To obtain event‐related EEG data, triggers marking stimulus onset were automatically transmitted from E‐Prime 2.0 to the Scan 4.3 software during EEG recording. The left (*A*1) and right (*A*2) mastoids were used as online references [(*A*1 + *A*2)/2], and a ground electrode was placed on the midforehead on the Quick‐Cap. Additionally, two sets of bipolar electrodes were placed on the upper and lower sides of the left eye, and on the canthi of both eyes to monitor vertical (VEOG) and horizontal (HEOG) eye movements. Electrode impedances were kept below 10 kΩ. EEG data were acquired at a sampling rate of 1000 Hz per channel, filtered with a Butterworth bandpass filter (0.1–70 Hz) and a 60‐Hz notch filter, and recorded continuously onto a hard disk for subsequent offline analysis.

### 2.7 Data Reduction and Statistical Analyses

#### 2.7.1 Behavioral Data

Behavioral performance, including RTs and accuracy, was recorded using E‐Prime 2.0 software. RT data were further analyzed by excluding nonresponse trials, error trials, and correct trials with latencies greater than two standard deviations above the mean (Wang and Tsai 2016).

#### 2.7.2 Time–Frequency Analysis of EEG

The EEG data was analyzed using the SPM12 software package for MEG/EEG (www.fil.ion.ucl.ac.uk/spm/) along with custom MATLAB scripts (Wang et al. 2020, 2017). Only EEG data corresponding to eligible behavioral responses and free from artifacts were included in the analysis, resulting in inclusion segments of 91.40 ± 3.10 for HSG and 91.05 ± 4.58 for LSG in the FP500 condition and 89.40 ± 4.68 for HSG and 88.80 ± 4.80 for LSG in the FP1500 condition. Large artifacts were identified in the continuous EEG data and a correction for eye blinks was applied. The eye blink peaks were derived from the VEOG and used to perform eye movement correction for all electrodes. The data were segmented into epochs from −1500 ms relative to the WS to 1500 ms relative to the IS. Trials containing artifacts with amplitudes exceeding ± 150 μV were removed from the analysis. Oscillatory power amplitude was computed using a continuous Morlet wavelet transform with a factor of 6 on single‐trial data within the frequency range of 2–50 Hz (Roach and Mathalon 2008). The averaged oscillatory power of each condition for each participant was rescaled by the baseline values from −300 to −100 ms relative to WS onset, and taking the log10 transform of this quotient (dB) (dB power = 10 × 10 [power/baseline]) allowed a direct comparison of results of interest across frequencies. Given that theta and beta power around central‐midline areas have been considered to be associated with levels of temporal or motor preparation (Cravo et al. 2011; Tzagarakis et al. 2010), we focus on the electrode on the central area (i.e., Cz) for further analysis. The log‐transformed changes in signal power relative to the baseline for each timepoint and frequency were used as the measures of interest for statistical analysis.

### 2.8 Statistical Analysis

A two‐way mixed‐design ANOVA was conducted to analyze behavioral performance, including accuracy and mean RTs for correct trials, using a 2 (*Group*: HSG and LSG) × 2 (*Condition*: FP500 and FP1500) factorial structure with repeated measures. Bonferroni adjustments were applied for multiple comparisons, and no violations of ANOVA assumptions were detected. The significance level was set at *p* < 0.05. All behavioral data analyses were completed in JASP (Love et al. 2019).

Time–frequency EEG analysis was conducted using the SPM12 software package for MEG/EEG in MATLAB (Yao et al. 2024). To assess event‐related changes in EEG across all frequencies and time windows, one‐sample *t*‐tests were conducted to compare EEG power differences before and after the onset of the WS. Additionally, independent *t*‐tests were used to examine group effects in the FP500 and FP1500 conditions. The significance level was set at *q* < 0.05, with a false discovery rate (FDR) correction applied to control for multiple comparisons (Benjamini and Yekutieli 2001).

To investigate the relationship between neural oscillations, behavioral data, and lower limb muscular strength, we conducted a mediation analysis using the PROCESS macro for R version 3.5.3. Prior to this, Pearson's correlation analysis was performed to assess the associations between lower limb muscular strength (i.e., MMT score), EEG oscillation (i.e., beta power), and task performance (i.e., RTs). Given the EEG findings (see Figure 3, lower panels), beta power was extracted from time–frequency regions that demonstrated stronger group effects. Specifically, for the FP500 condition, average values were obtained from 200 to 500 ms after WS onset in the 18–25 Hz range, and for the FP1500 condition, from 500 to 1000 ms after WS onset in the same frequency range. Following Hayes' (2017) method, a data‐driven mediation analysis was subsequently performed using the PROCESS macro if the following conditions were met: (a) lower limb muscular strength was significantly correlated with beta power, (b) lower limb muscular strength was significantly correlated with RT, and (c) beta power was significantly correlated with RT. The analysis was conducted separately for FP500 and FP1500.

## 3 Results

### 3.1 Participant Characteristics

The participants' demographic data are shown in Table 1. The independent *t*‐tests showed that none of these variables, including age, *t*(38) = −0.69, *p* = 0.50; sex, *χ* ^2^(1) = 2.51, *p* = 0.11; BMI, *t*(38) = 1.50, *p* = 0.14; MMSE, *t*(38) = 0.00, *p* = 1.00; MoCA, *t*(38) = −0.12, *p* = 0.89; BDI, *t*(38) = −0.75, *p* = 0.459; and education, *t*(38) = 0.79, *p* = 0.44, differed significantly between the two groups.

**TABLE 1:** Demographics of participants in each group.

	Higher strength group	Lower strength group
Age (years)	70.40 ± 5.15	71.43 ± 4.86
BMI	24.55 ± 3.10	23.07 ± 3.11
Gender (M:F)	12:8	7:13
BDI	3.40 ± 3.20	4.20 ± 3.55
MMSE	29.75 ± 0.79	29.75 ± 0.44
MoCA	27.95 ± 1.19	27.95 ± 0.44
Education (years)	13.90 ± 3.39	12.85 ± 4.91
MMT‐D	4.35 ± 0.49	3.18 ± 0.25
MMT‐ND	4.15 ± 0.37	3.05 ± 0.22
MMT‐Average	4.25 ± 0.38	3.11 ± 0.15
TUG (s)	6.07 ± 1.14	6.79 ± 0.88

Abbreviations: MMT‐Average = averaged score of both legs; MMT‐D = MMT dominant leg; MMT‐ND = MMT nondominant leg.

### 3.2 Lower Limb Muscle Strength

This study employed the MMT test to evaluate participants' lower limb muscle strength. Independent *t*‐tests indicated that the HSG exhibited significantly higher MMT scores compared to the LSG for the dominant leg, *t*(38) = 9.60, *p* < 0.001; nondominant leg, *t*(38) = 11.46, *p* < 0.001; and the averaged leg score, *t*(38) = 12.43, *p* < 0.001 (see Table 1).

### 3.3 Functional Mobility Related to Fall Risk

This study used the TUG test to assess participants' functional mobility. Independent *t*‐test revealed that the HSG performed the TUG test faster than the LSG, *t*(38) = −2.24, *p* < 0.031 (see Table 1).

### 3.4 Behavioral Performance

#### 3.4.1 Accuracy

The HSG showed 99.8 ± 0.7% in the FP500 condition and 99.7 ± 0.6% in the FP1500 condition, whereas the LSG showed 99.7 ± 0.6% in the FP500 condition and 99.4 ± 1.3% in the FP1500 condition. Given the presence of a ceiling effect, statistical analysis was not conducted for accuracy performance.

#### 3.4.2 Response Time

**FIGURE 2:**
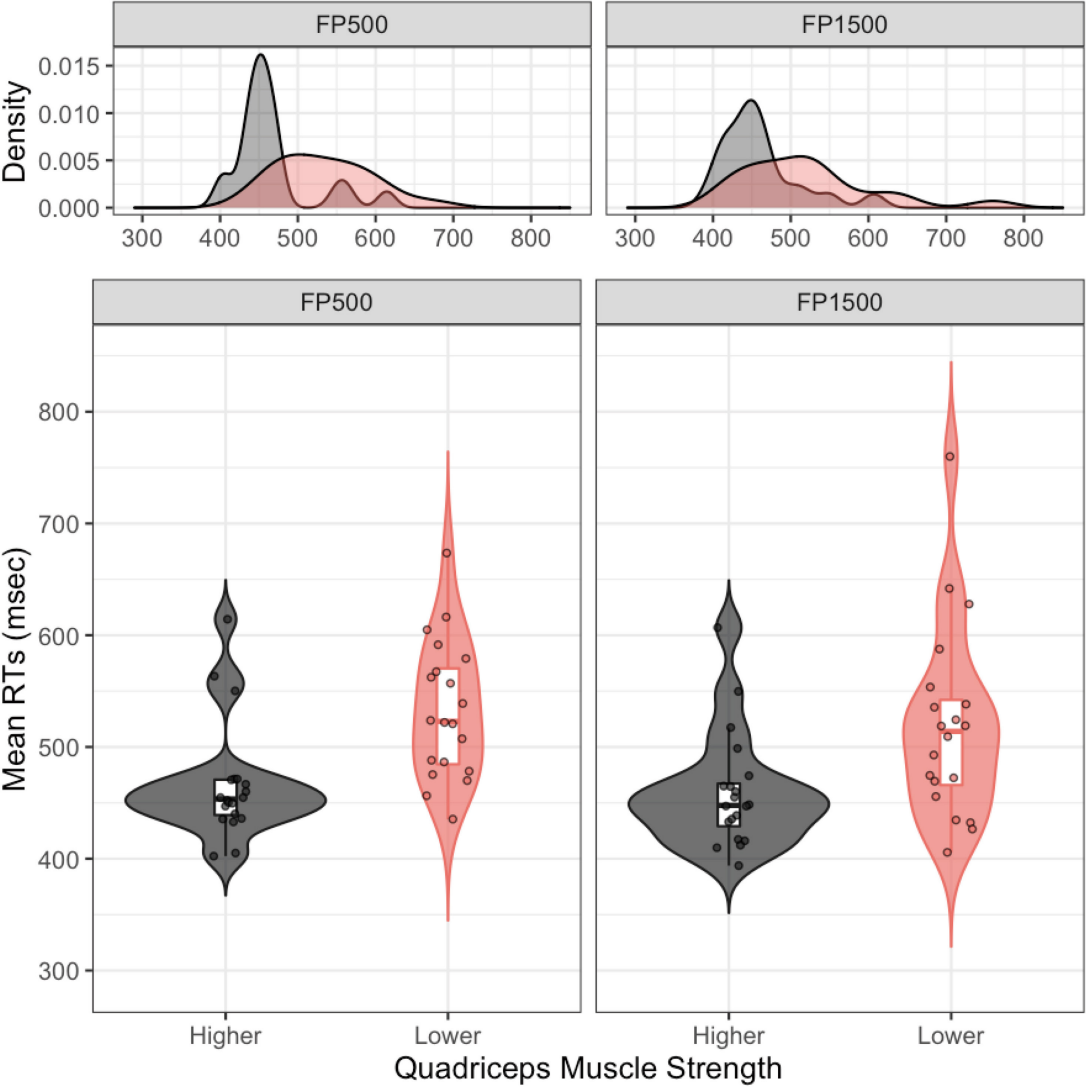
The mean RTs for elderly adults with high and low levels of lower limb muscle strength in the FP500 and FP1500 conditions are displayed in the figure. The violin plots show the distribution of mean RTs, including the mean (represented by the central dot in the box), median (represented by the central line in the box), the first and third quartiles (represented by the edges of the box), minimum and maximum (represented by the whiskers), and each individual participant (represented by dots).

Figure 2 and Table 2 present the mean RT for trials across different groups and conditions. A significant Group main effect was observed, *F*(1, 38) = 10.56, *p* = 0.002, *η*_p_^2^ = 0.22, with the HSG showing shorter RTs than the LSG. The *Group* by *Condition* interaction, *F*(1, 38) = 0.34, *p* = 0.566, *η*_p_^2^ = 0.01, and *Condition*, *F*(1, 38) = 3.06, *p* = 0.089, *η*_p_^2^ = 0.07, and were not significant, although RTs for the FP1500 condition were numerically shorter with a medium effect size than that of the FP500 condition.

**TABLE 2:** Response time performances of participants in each group.

Foreperiod (FP)	Higher strength group	Lower strength group
FP500	466.43 ± 51.98 ms	532.73 ± 61.04 ms
FP1500	459.52 ± 51.23 ms	518.99 ± 85.52 ms

### 3.5 Time–Frequency Analysis of EEG

**FIGURE 3:**
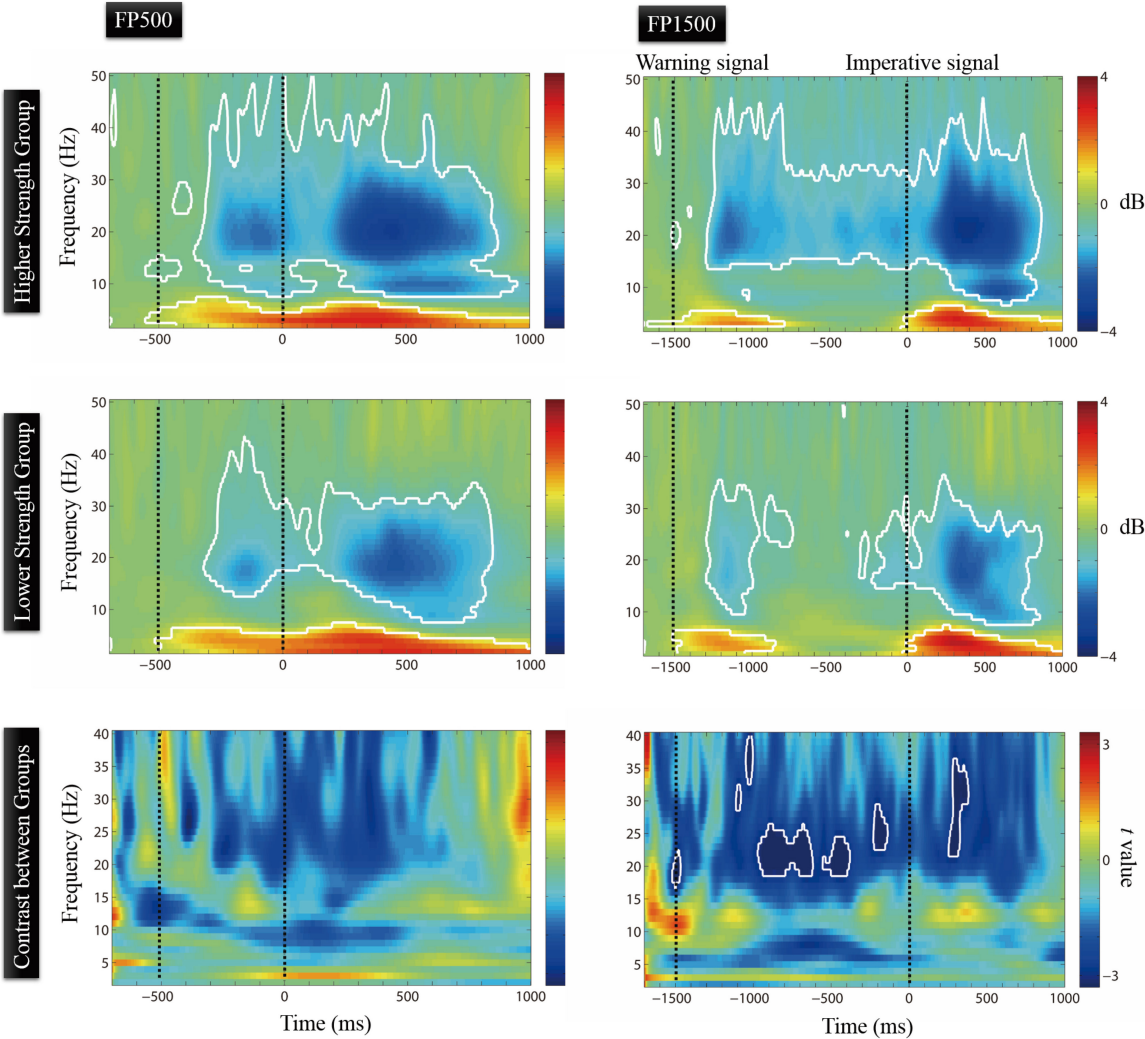
The results of the EEG analysis were shown in the time–frequency representation using Morlet wavelets over the Cz site. The upper and middle panels show the event‐related EEG oscillations in elderly adults with higher and lower levels of lower limb muscle strength, respectively. The WS presented at time zero (*t* = 0). The left panels show the results for the FP500 condition and the right panels show the results for the FP1500 condition. The lower panels show the contrast between the two groups for each condition, and the white line–enclosed regions represent temporal clusters that are statistically significant (at *q* < 0.05, FDR correction).

Figure 3 illustrates the event‐related changes in EEG power oscillations over the Cz site during the variable FP paradigm relative to the baseline interval (i.e., following the WS). The results indicate that both the FP500 and FP1500 conditions elicited similar patterns of increased theta (4–7 Hz) and decreased beta (15–35 Hz) bands following the onset of the WS (all *q*s < 0.05, FDR corrected). These patterns of oscillatory theta and beta activities were similar for both groups in the FP500 condition. However, in the FP1500 condition, we observed a sustained event‐related decrease in beta power throughout the preparatory period (all *q*s > 0.05, FDR corrected) in the HSG, whereas in the LSG, this decreased beta power weakened and eventually disappeared (all *q*s > 0.05, FDR corrected) before the upcoming IS. Specifically, a group comparison revealed that, during the later preparation period in the FP1500 condition, the HSG exhibited stronger beta desynchronization than the LSG (enclosed areas: around 18–25 Hz, 500–1000 ms after the WS onset, and around 20–35 Hz, 200–300 ms after IS; all *q*s < 0.05, FDR corrected). However, no such effect was observed in the FP500 condition (all *q*s > 0.05, FDR corrected). There were no significant differences between the groups for other frequency bands (all *q*s > 0.05, FDR corrected).

### 3.6 Correlations Between Lower Limb Strength, Beta Power, and RTs

**FIGURE 4:**
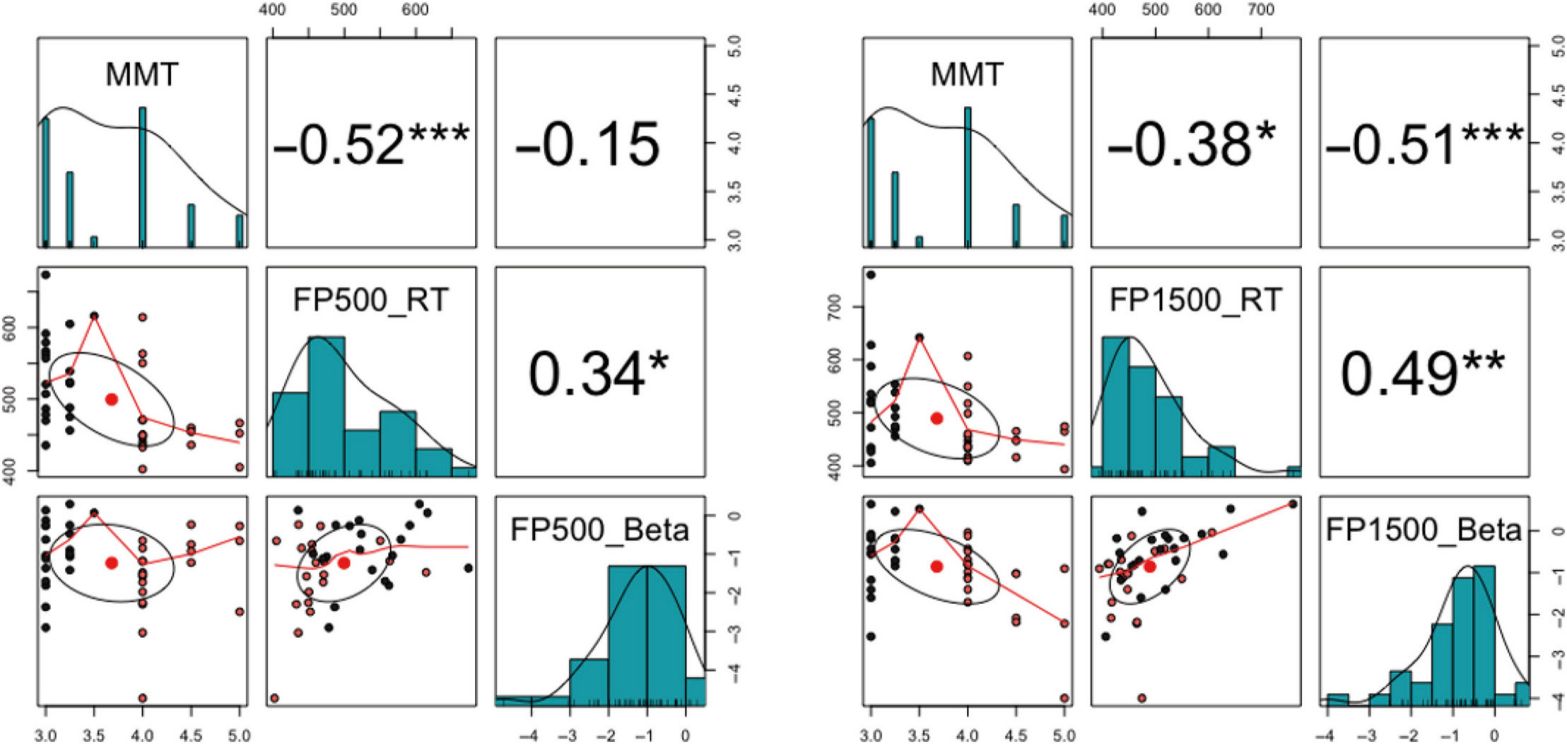
Scatterplot matrix illustrating the pairwise relationships between MMT, beta power, and mean RTs in the FP500 and FP1500 conditions. Each cell contains a scatterplot representing the relationship between two variables, with histograms displayed along the diagonal and correlation coefficients shown above the diagonal. In the scatterplots, red dots indicate high levels of lower limb muscle strength, whereas black dots represent low levels. The confidence ellipse reflects the likely range of data points based on the estimated correlation between the two variables; a narrower ellipse suggests a stronger correlation. *Note:* *p < 0.05, **p < 0.01, ***p < 0.001.

As shown in Figure 4, in the FP500 condition, MMT was significantly correlated with RT (*r* = −0.52, *p* < 0.001) but not with beta power (*r* = −0.15, *p* = 0.37). Additionally, beta power was found to be significantly correlated with RT (*r* = 0.34, *p* = 0.031). In the FP1500 condition, MMT was significantly correlated with RT (*r* = −0.38, *p* = 0.015) and beta power (*r* = −0.51, *p* < 0.001). Additionally, beta power was found to be significantly correlated with RT (*r* = 0.49, *p* < 0.001).

### 3.7 Mediation of Beta Power on the Correlation Between Lower Limb Strength and RTs

**FIGURE 5:**
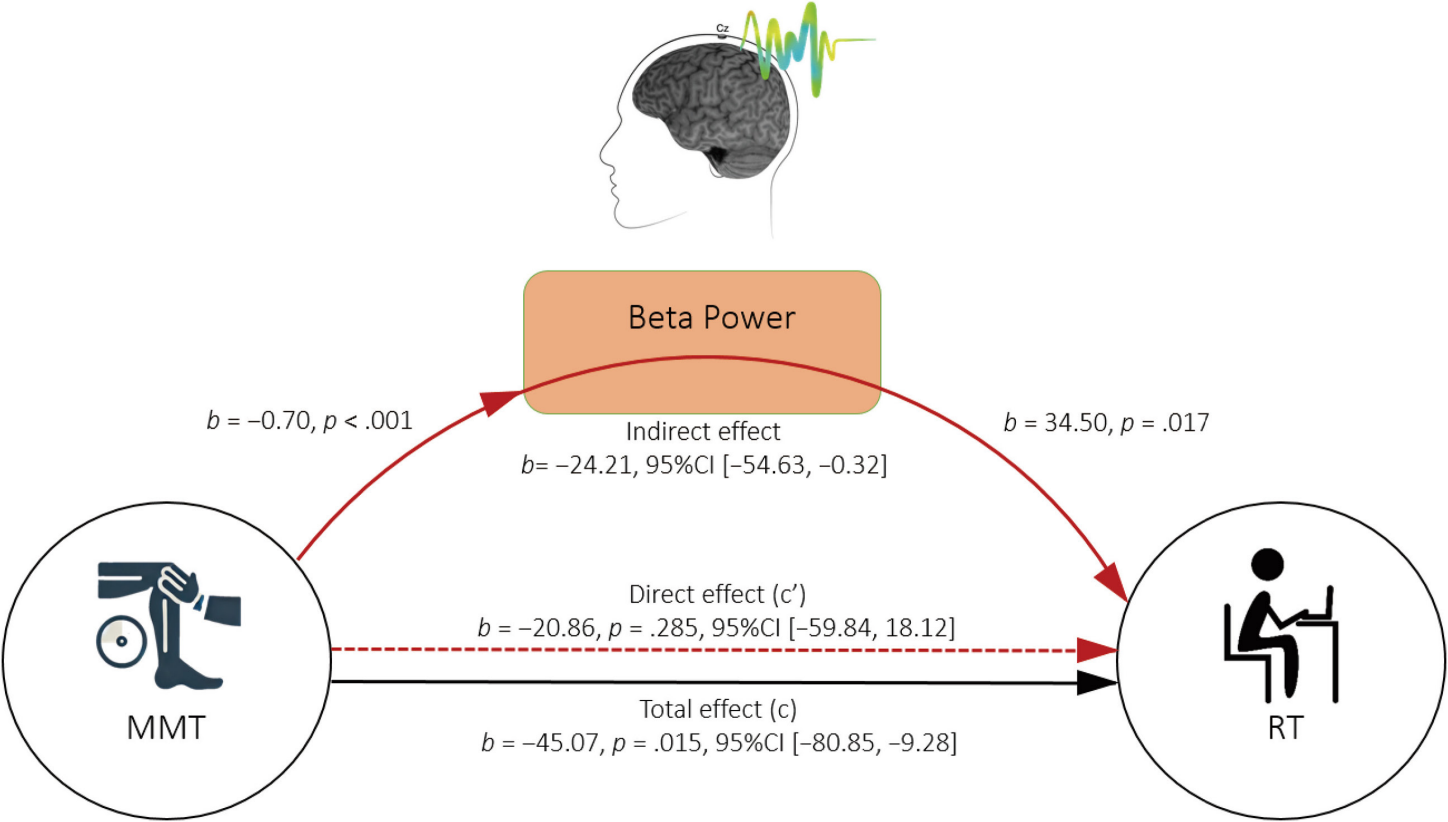
Mediation model illustrating the effect of beta power on the relationship between lower limb strength (MMT) and task performance (RT) in the FP1500 condition. The model depicts the mediation analysis between MMT and RT, with the beta power of EEG oscillation serving as the mediator. Arrows represent the direct and indirect pathways, indicating the influence of lower limb strength on task performance both directly and through beta power.

Because the correlation results indicated significant intercorrelations among lower limb strength, beta power, and RTs only in the FP1500 condition, the mediation analysis examining the effect of beta power on the relationship between lower limb strength and RTs was conducted exclusively for this condition. Following Hayes' (2017) bootstrapping method, a mediation effect is considered to be present if two conditions are satisfied: (a) The indirect effect (IE) of the independent variable (IV) on the dependent variable (DV) through the mediator must be significant. This IE can be determined either by the difference between the total and direct effects or by the product of path a (the effect of the IV on the mediator) and path b (the effect of the mediator on the DV). (b) The bias‐corrected 95% confidence interval (CI) for the IE, based on 10,000 bootstrap resamples, must not include 0. As illustrated in Figure 5, the mediation analysis revealed that beta oscillation fully mediated the relationship between lower limb muscle strength and mean RT in the FP1500, as indicated by a bootstrap CI that did not include 0 (coefficient = −24.21; 95% CI = [−53.51, −0.24]).

## 4 Discussion

The aim of the present study was to examine the effect of lower limb muscle strength on functional mobility related to fall risk and temporal preparation for lower limb responses during a variable FP paradigm, with concurrent EEG measurements used to explore the underlying neural mechanisms. The main findings indicate that elderly adults with greater quadriceps muscle strength exhibited not only better functional mobility, as evidenced by superior TUG performance but also faster RTs during the variable FP paradigm compared to their lower strength peers. Further, the time–frequency representation of EEG revealed that HSG demonstrated sustained oscillatory beta activity that was stronger than LSG only during the FP1500 condition but not the FP500 condition. Notably, beta power during the preparatory period was found to mediate the relationship between lower limb strength and RT in longer FP trials. These findings provide new insights into potential sources of impairment in lower limb response preparation related to the functional mobility associated with fall risk in elderly adults and highlight the positive effect of maintaining good lower limb strength in mitigating this risk.

Consistent with a large sample study showing a negative association between the occurrence of falls with quadriceps muscle strength (Ahmadiahangar et al. 2018), our findings indicate that older adults with greater quadriceps strength performed better on the TUG test compared to their lower strength counterparts. This suggests that maintaining higher levels of lower limb strength is associated with greater functional mobility, potentially reflecting a lower risk of falls. Therefore, our results highlight the importance of quadriceps strength as a key component of lower limb muscle strength in understanding the potential mechanisms underlying fall risk.

Considering that research has shown a relationship between quadriceps strength and cognitive performance in elderly adults (Chen et al. 2015; Scherder et al. 2010), we also examined whether the level of quadriceps strength modulates temporal preparation, particularly in making lower limb responses in a choice RT task. Specifically, we adopted a variable FP paradigm, where participants needed to monitor the changing conditional probability of the IS occurrence over time (Niemi and Näätänen 1981), a task that relies on frontal‐based cognitive function (Vallesi et al. 2007). Our findings revealed that elderly adults only exhibited a marginal FP effect, regardless of their lower limb strength level. This finding appears to partially align with a previous study that reported a larger variable FP effect in the elderly (Bherer and Belleville 2004), although it contrasts with the results of Vallesi et al. (2009), which demonstrated a trend for the opposite pattern of the FP effect in elderly adults. Differences in experimental design, such as the use of lower limb responses in the present study and the manipulation of stimulus occurrence probability during the short FP (e.g., 33% vs. 50%) across different studies, may explain the observed discrepancies. Nevertheless, findings from our study, along with earlier research, suggest that elderly adults may be less efficient at monitoring changes in probability over time and may struggle to maintain an optimal level of preparation across the entire range of FPs.

Interestingly, the group difference in behavioral findings revealed that elderly adults with higher quadriceps strength responded faster overall on the task than their lower strength peers. It appears that both groups attempted to anticipate the timing of the IS using the temporal dynamics of conditional probability; however, the longer RTs suggest that the LSG had inferior processing speed for lower limb responses in this context. The lack of a group‐by‐condition interaction might be attributed to the weak FP effect observed in both groups. Although this absence of interaction limits our ability to determine whether lower limb strength is specifically related to the ability to rapidly establish or maintain optimal preparatory levels, it may instead reflect a general age‐related slowing effect observed across various tasks. Previous research has suggested that age‐related cognitive slowing is linked to the vulnerability of frontal white matter to age‐related decline (Eckert 2011). For example, Chee et al. (2009) observed that processing speed showed a significant positive correlation with gray matter volume in several frontal areas in a sample of 248 individuals aged 55–86 years. Given that the cognitive ability to monitor temporal changes in the conditional probability of an upcoming IS under varying FP on a trial‐by‐trial basis relies on frontal functions (Vallesi et al. 2009; Vallesi and Shallice 2007; Vallesi et al. 2007), our findings suggest that older adults with greater quadriceps strength may possess superior frontal functioning, resulting in enhanced processing speed in a variable FP paradigm. Notably, a meta‐analysis has identified an association between muscle function and specific regional gray matter volumes, including the bilateral ventromedial prefrontal cortex (Kilgour et al. 2014). Indeed, our results align with previous studies linking quadriceps strength to higher‐order cognitive function (e.g., executive function) in older adults (Chen et al. 2015; Scherder et al. 2010). Therefore, a cognitive task incorporating variable FP appears to be a suitable method for investigating the relationship between physical fitness and its impact on cognitive aging, even in the context of a choice RT task.

Time–frequency analysis of EEG was employed to explore the underlying mechanisms of temporal preparation associated with the observed behavioral findings. The results showed a significant event‐related increase in theta power and a decrease in beta power during the execution of the variable FP paradigm in both groups. Previous studies have suggested that theta oscillation synchronization serves as an attentional mechanism for temporal preparation (Buzsaki and Draguhn 2004). Supporting this, Cravo et al. (2011) found that by manipulating the prior probability of FPs, higher theta power was associated with FPs of lower temporal uncertainty. Thus, the modulation of theta oscillations observed here may reflect the attentional process with regard to the temporal expectation.

However, the group comparison did not reveal significant differences in theta power across time windows in the two FPs between elderly adults with varying levels of quadriceps strength, suggesting that this may not be the primary mechanism underlying the observed behavioral differences. Instead, we found that the LSG was unable to sustain the decreased oscillatory beta power during the FP1500 trials. Additionally, the between‐group comparison revealed that the HSG exhibited stronger beta modulation than the LSG during the late time window of the preparation period in this longer FP condition. Given that an event‐related decrease in beta power has been associated with motor preparation during response uncertainty (Tzagarakis et al. 2010; Wang, Liang, et al. 2015), it is possible that individuals with poor quadriceps strength may struggle to maintain optimal motor preparation for a lower limb response during longer FP, leading to slower processing speed. This finding appears to extend previous behavioral studies investigating the effect of aerobic fitness on response preparation (Renaud, Bherer, et al. 2010; Renaud, Maquestiaux, et al. 2010). Specifically, elderly adults with higher levels of aerobic fitness, or those who improved their aerobic fitness through exercise interventions, exhibited shorter RTs during longer FPs and demonstrated a better ability to maintain preparatory levels over longer temporal periods. Finally, one possible explanation for the absence of a group difference in beta power during the short FP condition could be that both the HSG and LSG were able to establish their motor preparation immediately after the onset of the WS, and the LSG could maintain this preparatory state for up to 500 ms, as evidenced by the time–frequency EEG representation. Therefore, this duration may have been too short to detect any significant effect. Taken together, the current EEG results suggest that maintaining a higher level of quadriceps strength may enhance the ability to sustain motor preparation for lower limb responses in elderly adults, potentially reflecting a compensatory mechanism for optimizing motor responses, given their inefficient use of temporal information.

Interestingly, we observed significant correlations between lower limb strength, RT performance, and beta power during temporal preparation specifically in the longer FP condition (i.e., FP1500). To further investigate this, we explored the role of EEG oscillations in the relationship between lower limb strength and RT in the FP1500 condition. Mediation analysis indicated that beta power mediated the relationship between lower limb strength and RT, suggesting that the effect of lower limb strength on RT may be partially explained by its influence on beta power. This finding is particularly noteworthy, as previous research has demonstrated that other brain measures, such as the amplitude of the P3 component (Wang et al. 2016) and the size of hippocampal volume (Chaddock et al. 2010) can mediate the relationship between aerobic fitness and cognitive performance. Similar to these findings, our results extend the current literature by showing that greater lower limb strength is associated with stronger preparatory‐related modulation of beta power, which may, in turn, facilitate faster lower limb responses when temporal uncertainty is low. These results highlight the importance of maintaining higher levels of lower limb strength for enhanced brain activity during cognitive processing, which, in turn, supports better task performance in elderly adults. However, such inter‐correlations among lower limb strength, EEG oscillations, and task performance were not observed in the shorter FP condition (i.e., FP500). The absence of a relationship between lower limb strength and beta power in the shorter FP condition may be due to the insufficient duration of the FP period, which might prevent the establishment of an appropriate preparatory state and the activation of evident beta activity.

### 4.1 Limitations

Although the current findings provide novel EEG evidence on the relationship between lower limb muscle strength and temporal preparation in elderly adults, several limitations should be acknowledged. First, this study employed a clinical approach to indirectly measure the functional mobility associated with fall risk. However, the TUG test has been suggested to have limited predictive ability for falls in community‐dwelling older adults (Barry et al. 2014), and its utility may vary depending on an individual's cognitive and physical functioning (Schoene et al. 2013). Therefore, combining the TUG test with other assessment tools may be necessary for a more comprehensive evaluation of fall risk. Second, this study utilized a cross‐sectional design, limiting our ability to attribute individual differences in temporal preparation to specific lifestyle factors or genetic predispositions. Additionally, the use of a median split approach does not establish an absolute cutoff that would generalize to the broader population. Therefore, the generalizability of our findings remains uncertain and should be further validated in larger, more representative samples in future research. Third, it is worth mentioning that RT encompasses both premotor and movement time, it would be beneficial to examine the effects of lower limb muscle strength on these two components separately. Indeed, previous research has demonstrated that temporal preparation may influence movement time, as evidenced by electromyographic (EMG) data (Tandonnet et al. 2003). Therefore, incorporating EMG in future studies could be a promising approach to further disentangle the contributions of cognitive and motor processes to RT performance. Fourth, including a younger cohort for comparison could be a promising approach to examining whether lower limb muscle strength mitigates age‐related differences in temporal preparation. Finally, this study only measured nonspecific temporal preparation, limiting our ability to generalize the findings to contexts involving specific preparation. Although our results showed that elderly adults were less efficient in utilizing implicit temporal cues regardless of their physical fitness, it would be beneficial to extend these findings by investigating their preparation abilities when explicit temporal cues are provided.

### 4.2 Conclusion

This study contributes to the growing body of evidence suggesting that maintaining lower limb muscle strength during late adulthood may serve as a protective factor against declines in response preparation and functional mobility related to the risk of falls. Notably, our findings revealed that elderly adults did not exhibit a clear variable FP effect, indicating the ineffective use of temporal information to support lower limb responses in aging populations. Nevertheless, we observed that higher levels of lower limb strength were associated with more effective task‐related EEG modulation, which, in turn, was linked to improved task performance. This finding implies a potential compensatory mechanism through which greater lower limb strength may help preserve central motor preparation to facilitate faster lower limb responses despite age‐related challenges.

## Author Contributions

**Ming‐Cho Ho:** conceptualization, formal analysis, project administration, writing – original draft. **Hao‐Lun Fu:** formal analysis, visualization, writing – review and editing. **Shih‐Chun Kao:** conceptualization, writing – review and editing. **David Moreau:** conceptualization, visualization, writing – review and editing. **Wei‐Kuang Liang:** funding acquisition, methodology, resources, software. **Hsin‐Yu Kuo:** conceptualization, data curation, project administration, resources, writing – review and editing. **Chun‐Hao Wang:** conceptualization, formal analysis, funding acquisition, methodology, supervision, visualization, writing – original draft, writing – review and editing.

## Conflicts of Interest

The authors declare no conflicts of interest.

### Peer Review

The peer review history for this article is available at https://www.webofscience.com/api/gateway/wos/peer‐review/10.1111/ejn.70101.

## Data Availability

In accordance with HREC guidelines, data from this study may not be shared with other researchers without obtaining written reconsent from participants.
